# Student Vitality, Teacher Engagement, and Rapport in Studio Music Instruction

**DOI:** 10.3389/fpsyg.2020.01007

**Published:** 2020-05-21

**Authors:** Jennifer Blackwell, Peter Miksza, Paul Evans, Gary E. McPherson

**Affiliations:** ^1^Music Department, University of Hawai’i at Ma¯noa, Honolulu, HI, United States; ^2^Jacobs School of Music, Indiana University Bloomington, Bloomington, IN, United States; ^3^School of Education, University of New South Wales, Kensington, NSW, Australia; ^4^Melbourne Conservatorium of Music, The University of Melbourne, Parkville, VIC, Australia

**Keywords:** vitality, rapport, studio instruction, self-determination theory (SDT), music performance

## Abstract

Vitality is the feeling of being alive, vigorous, and energetic, and is an important indicator of overall motivation and wellbeing. Studio music instruction holds rich potential for creating feelings of vitality through close relationships, the potential for developing skills, and a shared endeavor of artistic expression. But they also have the potential to deplete vitality – through controlling teaching, a poor quality relationship, or harsh criticism from the teacher. The purpose of this study was to investigate relationships among student and teacher behavior, rapport, and students’ experiences of subjective vitality in the context of university-level applied performance lessons. Participants were six undergraduate instrumental music majors and their teachers located at universities in the United States and Australia, who were selected because they provided the highest (three participants) and lowest (three participants) scores on a measure of subjective vitality completed immediately following a studio music lesson. A lesson was recorded for each student-teacher participant pair, coded for the frequencies of 35 lesson behaviors, described with a qualitative contextual commentary, and rated for evidence of rapport and physical proximity. Clear differences emerged between the high and low vitality lessons with regard to questioning, feedback, modeling, student performance, and student talk. Teachers of high vitality students spent most or all of the lesson within close proximity to their student, and showed stronger rapport than teachers of low vitality students. The findings suggest that students’ vitality may depend on important differences in styles of teacher-student engagement and the quality of student-teacher relationships.

## Introduction

Studio music instruction is an integral feature of most higher education music programs. Advanced study of an instrument is challenging and progress can be slow, demanding a good deal of effort and delayed gratification for long-term rewards (Kennell, 2002). Additionally, studio music instructors are often influential figures for music students, as they have a more prolonged and intense relationship when compared to almost any other student-teacher dynamic in higher education (Nerland and Hanken, 2002). Thus, it is important to understand the types of practices that help teachers and students to develop a productive relationship. In particular, it is important to investigate what sorts of teaching behaviors would be related to students feeling energized and inspired to carry on with their studies. Such energy is important for developing the motivation to persist through and surpass the inevitable challenges one faces when working toward high level performance skills.

### Vitality

Ryan and Frederick (1997) proposed the psychological construct “subjective vitality” to describe “one’s conscious experience of possessing energy and aliveness” (p. 530). Subjective vitality is a phenomenological state that is theoretically tied to Ryan and Deci’s (2017) self-determination theory, a metatheoretical perspective that describes how and why people strive to experience psychological wellbeing and establish an identity aligned with their sense of self. Self-determination theory also describes how social conditions can either support or thwart human flourishing according to the degree to which they serve to help individuals fulfill the basic psychological needs of competence, autonomy, and relatedness (Ryan and Deci, 2017). Satisfaction of the basic psychological needs has been shown to be positively related to vitality, whereas frustration of basic needs is unrelated or negatively related (e.g., Baard et al., 2004; Ryan and Deci, 2008). For example, Ryan and Deci (2017) have documented how a student’s sense of vitality (along with other important motivational dispositions) could suffer if they perceive their school environment as controlling as opposed to autonomy-supportive. Although rarely studied in music, Miksza et al. (2019) found university level music majors’ reports of vitality were positively related to their ability to adapt to challenges and the quality of their relationships with their peers, whereas they were negatively related to their general experiences of stress. However, the ways in which university-level studio music instructors may be able to support or thwart their students’ sense of vitality through features of their instruction specifically has yet to be explored.

### Expert Studio Music Instruction

The master-apprentice relationship found in studio music instruction has been described as both a “secret garden” or “secret activity” that occurs behind closed doors, and a form of teaching that is too often based on an authoritarian model that promotes undue emphasis on a master musician’s model (see review, Hyry-Beihammer, 2010). As McMillan (2008) notes, studio music instructors often do not have formal qualifications in teaching, registration with professional associations, or ongoing professional development, and are likely to teach as they were taught in their own lessons. Burwell’s (2013) theoretical investigation explored the practice of studio music instruction as an example of the apprenticeship model. Apprenticeship has been a traditional form of education in many fields, particularly when “skills rather than propositional knowledge are to be cultivated” (Burwell, 2013, p. 279).

Much of the research aimed at investigating studio music instruction expertise to date has been focused on categorizing teachers’ time usage (Sogin and Vallentine, 1992; Siebenaler, 1997; Colprit, 2000; Creech, 2012), identifying a taxonomy of teacher behaviors that contribute to students’ skill acquisition by observing renowned pedagogues (Duke and Simmons, 2006; Duke and Chapman, 2011; Parkes and Wexler, 2012; Blackwell, 2018), and exploring the nature of interpersonal relationships between students and teachers (Nerland and Hanken, 2002; Gaunt, 2008, 2011; Montemayor, 2008; Clemmons, 2009/2010; Hyry-Beihammer, 2010; Creech, 2012; Schiavio et al., 2019). Studies categorizing time usage during studio music instruction have found that most of the time was spent on student and teacher performance, followed closely by teacher verbalizations (Sogin and Vallentine, 1992; Colprit, 2000; Creech, 2012). These studies are instructive in understanding how teachers use their time, and provide a basis for exploring why teachers might make those decisions, and in particular their impact on student outcomes.

Applying a more nuanced approach, Duke and Simmons (2006) sought to develop narrative descriptions of studio music instruction behaviors that are related to changes in student performance. Their analyses resulted in the identification of 19 pedagogical elements that they observed consistently across several lessons from three renowned teachers. These 19 elements were grouped into three categories: goals and expectations (e.g., choosing appropriate repertoire and lesson targets, maintaining consistently high expectations), effecting change (e.g., strategic starts/stops, repetition, pacing, timing breaks), and conveying information (e.g., specific negative feedback, instances of high-magnitude positive feedback, modeling, a focus on interpretive effects). The researchers proposed that these elements “comprise the highest form of instructional skill in music,” suggesting that the consistent teacher behavior they observed in the lessons was emblematic of the best available pedagogy in university-level studio music instruction (Duke and Simmons, 2006, p. 16).

Several researchers have since worked to extend Duke and Simmons’ (2006) findings by applying quantitative coding schemes (Parkes and Wexler, 2012; Blackwell, 2018) and broadening the student population to include pre-university level students (Duke and Chapman, 2011; Blackwell, 2018). Parkes and Wexler’s (2012) partial replication of Duke and Simmons’ (2006) study revealed many of the same pedagogical elements, however, nearly one third of the behaviors they identified were inconsistent with Duke and Simmons’ categorizations. For example, the teachers in Parkes and Wexler’s (2012) study more frequently provided positive feedback, allowed students to progress even when their performance was not completely accurate, often engaged in side-coaching while students were playing, conducted their lessons with a relatively slower pace, and allowed students to speak about their frustrations and ask questions. The researchers suggested that the differences between the studies may be have been due to differences in student ability, because the students in their study were less skilled than those in Duke and Simmons’ (2006) study (Parkes and Wexler, 2012).

Duke and Chapman’s (2011) and Blackwell’s (2018) extensions of Duke and Simmons’ (2006) research also found that teacher behavior seems to be dependent on student skill level. The expert pedagogue in Duke and Chapman’s (2011) study emphasized teaching pre-university violin students how to practice more than university-level students, suggesting his awareness of the need for younger students to develop independence. Blackwell (2018) examined the teaching behavior of violin and saxophone pedagogues who were renowned for their work with pre-university, undergraduate university level, and graduate university level students alike. Most of the elements identified in Duke and Simmons’ (2006) study were observed by Blackwell (2018), and the discrepancies that emerged were similar to those found by Parkes and Wexler (2012) (e.g., presence of side-coaching, not all student errors eliciting stops). However, Blackwell (2018) also noted that the teachers were often within close physical proximity to their students and found that the teachers’ behavior seemed to vary as a function of student age, with more modeling, teacher gesturing, and side-coaching occurring during lessons of younger students. This research suggests that expert teachers adapt their teaching in context-dependent ways to meet the needs of their students; additional research is necessary to understand the impact of these practices as perceived by students.

### Relationships and Rapport in Studio Music Instruction

Researchers who have investigated specific behavioral elements that elicit performance improvement have contributed much to our understanding of studio music instruction expertise. It is also important, however, to explore how productive interpersonal relationships and positive rapport between students and their teachers might be fostered. Nerland and Hanken (2002) have gone as far as suggesting that the intimate relationships forged in studio music instruction are like a parental bond, stating that “Working with the music implies that both student and teacher must expose themselves emotionally, and therefore they grow closer to each other on a personal level” (p. 180). Along similar lines, Clemmons (2009/2010) studied four master vocal teachers known for their abilities for “building strong relationships with their students” (p. 257). Data from interviews, observations, and surveys suggested that the teachers’ expertise was rooted in the following characteristics: establishing feelings of safety and respect to give students the security essential for developing positive relationships; setting clear expectations and relational boundaries to help students to feel successful; and maintaining an enthusiastic, positive teaching style to help students feel confident and enthusiastic about their learning.

Creech (2006) identified six relational types in her extensive study of 263 violin teachers, students, and parents: solo leader, dominant duo, dynamic duo, double duo, discordant duo, discordant trio, and harmonious trio (Creech, 2009). Case studies of teachers exemplifying each relational type illustrated how students of teachers who embodied the harmonious trio style achieved positive outcomes most consistently, explaining that this relational style was characterized by reciprocal communication, mutual respect, student-centered goals, and support for student autonomy (Creech, 2006). Similarly, Hyry-Beihammer (2010) describes a master piano teacher from Finland who maintains a collaborative relationship with students and adjusts their teaching to fit to students’ needs. Montemayor’s (2008) case study of a renowned flute teacher revealed similar findings, but also highlighted how a music studio can exist as an ecological system with a unique cultural context and value system between teacher and students. In addition to developing performance skills, Montemayor (2008) describes how the flute teacher created a sense of relatedness and support among her students that ultimately contributed to them forming an identity as a flutist.

As far as can be determined, Blackwell’s (2018) aforementioned study of two artist level studio music instructors working with students of various ages is the only example of a quantitative research investigation that examines empathy as a contributor to a teacher’s ability to develop interpersonal relationships (i.e., the Toronto Empathy Questionnaire; Spreng et al., 2009). Interestingly, the teachers in her study both scored at least one standard deviation above published scale norms, suggesting that higher levels of empathy may be associated with their teaching expertise. Moreover, the teachers emphasized developing rapport with students, providing honest feedback, and fostering a culture of supportive peers as critical components of their teaching during interviews.

While there have been efforts made toward examining the intricacies of interpersonal relationships in applied teaching (Nerland and Hanken, 2002; Kurkul, 2007; Montemayor, 2008; Gaunt, 2008, 2011; Wexler, 2008; Clemmons, 2009/2010; Hyry-Beihammer, 2010; Creech, 2012; Nolan, 2012; Biasutti and Concina, 2018; Blackwell, 2018), theoretically grounded constructs of rapport are notably absent in the literature. Tickle-Degnen and Rosenthal (1990) provide a useful model for investigating rapport, describing it as a dynamic relationship between three interrelated components: mutual attentiveness, positivity, and coordination. Mutual attentiveness refers to the cohesiveness among participants during interactions, including the expression of mutual attention to and involvement with one another. When a pair is mutually attentive, their focus is directed toward the other person and they experience intense mutual interest. Positivity is described as the feeling of mutual friendliness and caring. Although positivity is closely related to mutual attentiveness, a high level of one does not necessarily ensure a high level of the other – for example, a verbal fight would constitute high mutual attentiveness without positivity (Tickle-Degnen and Rosenthal, 1990). Coordination is conceptualized as balance, harmony, and being “in sync” such that actions between individuals have a sense of regularity and predictability that results in smooth interactions. Taken together, these three components provide a multidimensional characterization of rapport that could be useful as criteria for observing and evaluating rapport in student-teacher relationships.

Some researchers have suggested that rapport could be developed through non-verbal behavior (Kurkul, 2007) and constructs such as immediacy, which has been defined as “those communication behaviors manifested and perceived when a person maintains closer physical distance” (Andersen, 1978, p. 17). Immediacy includes a variety of nonverbal behaviors that reduce distance between people, either by decreasing physical distance or the less tangible psychological distance, thus communicating a sense of closeness (Andersen et al., 1981; Baringer and McCroskey, 2000). Roseth (2018) surveyed secondary band and orchestra teachers and found that female teachers and beginning ensemble teachers reported higher levels of immediacy behaviors than male teachers and those who worked with more advanced ensembles.

In a follow up experiment, Roseth (2018) found that students’ perception of teacher immediacy was positively correlated with student affect, motivation, and group cohesion; a finding that suggests that immediacy might help to explain differences in the quality of student-teacher relationships. Proximity and immediacy have also been found to be related to learning outcomes in a curvilinear manner in teaching contexts, such that relatively low and high perceptions of immediacy proximity are related to weaker outcomes, whereas moderate levels of proximity are related to optimal outcomes (Comstock et al., 1995; den Brok et al., 2004). Although immediacy behaviors can include physical contact, facial expressions, vocal inflection, posture, and other movements (Baringer and McCroskey, 2000, p. 178), we focus on physical proximity as an element of immediacy in the present study.

### Aims of the Study

Although research dealing with studio music instruction has yielded much insight into the components of pedagogical expertise, relatively few studies have been conducted that explicitly connect these components with formal constructs representing student/teacher rapport and students’ vitality. Research that identifies teaching practices that are beneficial for performance skill acquisition might also lead to the cultivation of productive interpersonal relationships and optimal student affect would be valuable for teachers and researchers alike. As such, the purpose of this study was to investigate relationships among student and teacher behavior, rapport, and students’ experiences of subjective vitality in the context of university-level applied studio music instruction. Specifically, we sought to clarify relationships between teaching behaviors emblematic of expert teaching and (1) student’s experiences of subjective vitality, (2) ratings of student-teacher rapport, and (3) ratings of teacher proximity.

## Materials and Methods

### Participants

Participants were six undergraduate instrumental music majors and their teachers located in prominent university music programs in the United States and Australia. They were sampled from a larger study of motivation and self-regulated learning in studio music instruction, and selected on the basis of having the highest and lowest scores on a measure of subjective vitality (see below). Four of the student participants were female and two were male; they played cello, flute (two students), F horn, trumpet, or trombone. The student participants reported having spent 6 to 14 years taking formal private lessons. The 6 university-level teacher participants have international reputations as performers and pedagogues, reported a range of 18–56 years teaching in prominent music schools/conservatories, and had spent 11–55 years as professional performing musicians. Four of the teacher participants were male and two were female. Informed consent to participate in this study was obtained from both the students and their teachers.

### Subjective Vitality

The six student participants were purposively selected for this study from a sample of 125 potential participants who were video recorded taking a lesson with a university-level teacher. The mean age of the full sample was 20.84 years (*SD* = 2.66), with 50.4% female, 39.2% male, and 0.9% electing not to share their sex (9.6% of respondents skipped this question). We selected these six students as participants for the present study because they represented the three highest and lowest scores on an adaptation of Ryan and Frederick’s (1997) measure of subjective vitality. We chose to limit the number to six participants because they represented the most extreme scores available in the sample, and thus provided maximum contrast. We adapted the original subjective vitality scale (Ryan and Frederick, 1997) so that the items were worded to reflect the students’ experiences immediately following a lesson that just ended. The students rated each of the following items on a 7-point, Likert-type scale ranging from “1-Not at all true to me” to “7-Very true of me” according to their feelings immediately after their lesson: “When the lesson was finished, I felt alive and vital”; “After the lesson, I felt so alive I just wanted to burst”; “After the lesson, I had energy and spirit”; “I am looking forward to each new day”; “After the lesson, I felt alert and awake”; and “I feel energized after the lesson.” The seventh, negatively worded item from Ryan and Frederick’s (1997) scale was omitted as per recommendations made by Bostic et al. (2000). Internal consistency of this measure as evaluated with all 125 possible participants’ responses was excellent, Cronbach’s alpha = 0.92, consistent with previous research on the scale (Ryan and Frederick, 1997; Bostic et al., 2000). In other studies of subjective vitality, the scale has validational correlates with valence and arousal, as well as predictive validity based on factors expected to increase subjective vitality such as physical activity, social interaction, being outdoors, and interactions with nature (Ryan and Deci, 2017). The three highest (*M* = 6.94, min = 6.83, max = 7.00) and three lowest (*M* = 2.17, min = 1.78, max = 2.83) scoring participants were included in this study. All six participants had different studio music instructors, and both research sites were represented in the high and low vitality groups.

### Video Recording Procedures

The participants were recorded taking a lesson with their regular studio music instructor at their usual time in their teacher’s studio space. A research assistant attended lessons at teachers’ studios to set up the video camera (Zoom Q4n video recorder), left for the duration of the lesson, and then returned to collect the equipment. The camera angle for each lesson was set in consultation with the teacher to capture video of both participants so that it would be possible to document the interactions between the teacher and student. The research assistant provided either a link to the online version of questionnaire or a paper version of a questionnaire containing demographic questions and the subjective vitality measure for the students to complete immediately following the lesson. The video recordings were then transferred to a secure cloud drive for analysis.

### Video Recording Measures

#### Lesson Behaviors

The lesson videos were coded for the presence of teacher and student behaviors shown to be salient to studio music instruction expertise in previous research (Colprit, 2000; Duke and Simmons, 2006; Duke and Chapman, 2011; Parkes and Wexler, 2012; Blackwell, 2018). The teacher strategies we defined included a variety of types of questioning, discussion, feedback, modeling, and side coaching activities. We also coded whether a teacher reduced the complexity of a student performance task and behaviors in which teachers made physical contact with students. Regarding student behaviors, we coded for instances of student performance as well as various types of student talk and student questions. Lastly, we coded for when either student or teacher laughed during the lesson. See Appendix A for a list of all behaviors and descriptions of each. We used the freely available Behavioral Observation Research Interactive Software (BORIS) program, v. 7.5.3 (Friard and Gamba, 2016) to tally frequencies (i.e., mark the start) of each target behavior. No durational estimate of behavior was made. To establish interrater reliability of the observational analyses, we chose a video from one low and one high vitality student not included in the main study to independently analyze. Due to challenges in maintaining consistency when coding these pilot videos independently, the six participants’ videos used in the main study were coded collaboratively by the first two authors. All disagreements in coding were negotiated until agreement on every instance of a behavior was achieved.

#### Proximity

Physical proximity between the student and teacher was evaluated using a single item, researcher-designed, 5-point Likert-type rating scale. The measure resulted in an overall evaluation of the physical distance between student and teacher in the lesson, completed immediately after the behavioral analysis. The first two authors rated each video based on their overall impressions of the lesson by selecting one of the following options: “5-Teacher is near the student all or nearly all of the time”; “4-Teacher is near the student more often than far”; “3-Teacher spends roughly equal time near to and far from student”; “2-Teacher is far from the student more often than near”; “1-Teacher is far from student all or nearly all of the time.” Five options were chosen because we felt they captured all of the reasonably identifiable options for proximity, given limitations of camera placement and differences in room sizes. Any disagreements in ratings were discussed until agreement was achieved.

#### Rapport

Student and teacher rapport evident during the video-recorded lessons was also measured via a researcher-designed measure. The rapport measure consisted of three Likert-type items derived from Tickle-Degnen and Rosenthal’s (1990) theoretical model of rapport. The first two authors rated each video by responding on a six-point Likert-type scale (i.e., “1-Strongly Disagree” to “6-Strongly Agree”) to the following items meant to operationalize Tickle-Degnen and Rosenthal’s dimensions of mutual attentiveness: “There is a sense of cohesiveness and unification between the teacher and student as a result of their mutual attention to the lesson in the moment,” positivity: “The interactions between the teacher and student are friendly and supportive,” and coordination: “The teacher and student act in sync with each other as though they are a team.” Any disagreements in ratings were discussed until agreement was achieved. The ratings for the three items were then summed to create a composite score with a possible range of three to 18 points.

#### Contextual Narratives

A qualitative contextual narrative characterizing each of the lessons was also completed during the coding process. While one of the authors operated the BORIS software, another took notes and worked toward completing a narrative designed to capture elements of the lesson that would otherwise not be readily apparent from the behavioral analysis. The first two authors discussed the categorization and resolved any disagreements that occurred. Categories for this analysis included tone of the lesson, goals and priorities of the lesson, general styles of student and teacher engagement, the sense of student progress due to instruction, and other. These descriptions were then analyzed by the first author to create an overall characterization of the high and low vitality lessons (see Appendix B).

## Results

### Behavioral Analyses

Overall, we observed and coded 35 distinct behaviors in the 6 video-recorded lessons. Because the sample size for this study precludes the use of inferential statistical analyses, we chose to present the results of our analyses by summarizing notable trends of pronounced differences between the groups with descriptive statistics only (see Tables 1–3). In most cases, greater frequencies of the behaviors emblematic of studio music instruction expertise were observed in the lessons of the high vitality students as compared to those of the low vitality students. Even though the assigned lesson time was 1 h for each student, on average, the low vitality students’ lessons were approximately 14 min shorter (*M* = 53 min 44 s) shorter than high vitality students’ lessons (*M* = 1 h 7 min 49 s) and the range of the high vitality student lessons (minimum = 52 min 4 s, maximum = 1 h 16 m 2 s) was wider than that for the low vitality students (minimum = 51 m 13 s, maximum = 57 m 44 s). Of course, it is not possible to preclude the possibility that lesson times were impacted by other factors. Nonetheless, working with someone whose vitality is depleted is probably not a pleasant experience, so future research may be interested in circumstances under which teachers may wish to end lessons earlier than what they had planned or scheduled for.

**TABLE 1:** Summary statistics of teachers’ verbal behaviors in low and high vitality students’ lessons.

	**Low vitality students**				**High vitality students**			
	**Mean**	**1**	**2**	**3**	**Mean**	**1**	**2**	**3**
**Teacher question – elaborate**	**3.67**	**3**	**3**	**5**	**16.67**	**3**	**1**	**46**
**Teacher question – check**	**4.67**	**8**	**1**	**5**	**14.67**	**25**	**2**	**17**
Teacher question – goals	1.67	2	3	0	0.33	0	1	0
**Teacher question – orienting**	**5.67**	**7**	**4**	**6**	**1.67**	**3**	**0**	**2**
Discussion – practicing	4.33	7	4	2	4.00	8	2	2
Discussion – personal, unrelated	1.00	2	0	1	1.67	1	2	2
**Discussion – personal, music**	**0.33**	**1**	**0**	**0**	**6.67**	**15**	**2**	**3**
Feedback – low recognition of general progress	6.33	3	6	10	6.00	5	8	5
Feedback – high recognition of general progress	2.00	0	4	2	2.33	4	2	1
**Feedback – low recognition of specific progress**	**2.00**	**5**	**1**	**0**	**11.67**	**30**	**4**	**1**
**Feedback – high recognition of specific progress**	**0.33**	**0**	**1**	**0**	**6.33**	**19**	**0**	**0**
Feedback – person-directed critique	3.67	1	9	1	3.33	9	1	0
Feedback – low person-directed praise	0.67	2	0	0	4.00	4	8	0
Feedback – high person-directed praise	0.00	0	0	0	0.33	1	0	0
**Feedback – behavior-contingent critique**	**26.33**	**7**	**35**	**37**	**54.67**	**132**	**17**	**15**
**Feedback – behavior-contingent praise**	**9.67**	**1**	**16**	**12**	**4.33**	**3**	**1**	**9**
Feedback – normative critique	0.33	1	0	0	0.33	1	0	0
Feedback – normative praise	0.33	0	1	0	0.00	0	0	0
**Feedback – instructional information**	**14.67**	**25**	**5**	**14**	**32.67**	**22**	**34**	**42**

**Bold face rows indicate differences discussed in text.**

**TABLE 2:** Summary statistics of teachers’ physical behaviors in low and high vitality students’ lessons.

	**Low vitality students**				**High vitality students**			
	**Mean**	**1**	**2**	**3**	**Mean**	**1**	**2**	**3**
**Modeling – instrument**	**49.33**	**34**	**56**	**58**	**39.00**	**20**	**68**	**29**
**Modeling – vocal**	**8.00**	**2**	**5**	**17**	**55.33**	**138**	**14**	**14**
**Modeling – cognitive**	**0.33**	**0**	**1**	**0**	**5.33**	**1**	**2**	**13**
**Gestural side coaching during performance**	**8.67**	**1**	**10**	**15**	**17.67**	**45**	**2**	**6**
**Vocal side coaching during performance**	**2.67**	**0**	**8**	**0**	**31.00**	**89**	**3**	**1**
Modify passage to reduce complexity	5.00	4	6	5	6.00	7	9	2
**Teacher touches student – physical manipulation**	**0.00**	**0**	**0**	**0**	**0.33**	**0**	**1**	**0**
**Teacher touches student – rapport**	**0.00**	**0**	**0**	**0**	**0.67**	**0**	**0**	**2**

**Bold face rows indicate differences discussed in text.**

**TABLE 3:** Summary statistics of low and high vitality students’ behaviors.

	**Low vitality students**				**High vitality students**			
	**Mean**	**1**	**2**	**3**	**Mean**	**1**	**2**	**3**
**Student performance**	**56.33**	**38**	**62**	**69**	**105.67**	**182**	**107**	**28**
**Student question – request for assistance**	**9.33**	**16**	**4**	**8**	**3.00**	**0**	**3**	**6**
**Laughing**	**10.33**	**11**	**10**	**10**	**26.00**	**32**	**1**	**45**
Student question – request for feedback	1.00	2	0	1	1.67	1	3	1
**Student talk – negative expression**	**12.33**	**32**	**1**	**4**	**5.67**	**0**	**14**	**3**
Student talk – positive expression	3.67	11	0	0	0.67	1	0	1
Student talk – off task	1.33	4	0	0	0.00	0	0	0
**Student comment – description**	**18.33**	24	10	21	**33.33**	13	20	67

**Bold face rows indicate differences discussed in text.**

#### Teacher Questions, Discussion, and Feedback (see Table 1)

The types of questions teachers asked in the two groups of lessons were quite different. On average, teachers of the high vitality students checked on students’ understanding and asked students to elaborate on topics much more frequently. One teacher asked 46 elaboration questions, which is substantially larger amount than the maximum of 5 elaboration questions observed among the low vitality students’ lessons. In contrast, teachers of the low vitality students needed to ask students more orienting questions, such as “what are you working on?” — suggesting they were less aware of their students’ current status and state of progress overall. The teachers of both groups of students discussed practicing and unrelated personal issues with similar frequency, however, teachers of high vitality students were more likely to discuss their own personal musical backgrounds during their teaching.

There were several clear differences in the types of feedback teachers provided in the low vs. high vitality students’ lessons as well. Teachers of high vitality students were more likely to recognize the progress students were making on specific tasks during their lessons, and did so more often than teachers of low vitality students with both low and high magnitude responses. Similar differences were observed in regard to behavior-contingent critique, in that teachers of high vitality students were much more likely to provide feedback that specified the task-related challenges the students were experiencing. Moreover, the maximum value for one high vitality students’ teacher’s instances of behavior-contingent critique was extremely high at 132. The high vitality students’ teachers were also more frequently observed providing general instructional information to assist students. In contrast, teachers of low vitality students were more frequently observed providing behavior-contingent praise.

#### Teacher Modeling, Side Coaching, and Touch (see Table 2)

Teachers of high vitality students’ lessons engaged in more instances of vocal and cognitive modeling (i.e., explaining the way they think/conceptualize a particular musical issue for the student), with maximum values of 138 and 13 instances, respectively. These values are extremely large compared to the maximum values of 17 instances of vocal modeling and 1 instance of cognitive modeling observed among the low vitality students’ lessons. However, on average, teachers of low vitality students modeled somewhat more frequently on their instruments (*M* = 49.33) as compared to teachers of high vitality students (*M* = 39.00).

The high vitality students’ teachers also exhibited much more gestural and vocal side coaching than the low vitality students’ teachers. Side coaching includes behaviors that encourage, remind, or support students as they perform, without pausing the performance. The difference in vocal side coaching was extreme with a maximum value of 89 instances among high vitality students compared to a maximum of 8 instances among low vitality students, although this trend was driven by one high vitality students’ lesson. Teachers of high vitality students also exhibited a few rare instances of touch, such as high fives or a pat on the shoulder, whereas none were observed among teachers of low vitality students.

#### Student Performance and Verbalizations (see Table 3)

On average, students reporting high vitality performed nearly twice as much (*M* = 105.67) as those reporting low vitality (*M* = 56.33), although one of the students reporting high vitality performed less instances (minimum = 28) than one of the students reporting low vitality (minimum = 38). Students reporting low vitality were more likely to ask for help during their lessons and express negativity (e.g., frustration, anger, sadness). In contrast, students reporting high vitality were more likely to comment during the lesson (e.g., answer questions, engage in discussion). Lastly, on average, more laughing occurred during the high vitality students’ lessons (*M* = 33.33) compared to those of the low vitality students (*M* = 18.33).

It is important to reiterate a key point, described in several instances above: the trends we highlight in terms of observed behaviors for the groups of low and high vitality students were not consistently seen for all students in the low or high vitality groups. For example, despite the fact that there were more orienting questions in the low vitality group’s lessons overall, there were more orienting questions asked of student two in the high vitality group than in either student two or three in the low vitality group. In other instances, a trend observed between the groups was driven by a relatively extreme amount of behavior demonstrated by one teacher. The 132 instances of behavior-contingent critique exhibited in one of the high vitality lessons drove up the mean such that it would appear high vitality teachers generally give more critiques of this kind, however, the other two high vitality lessons had fewer instances of behavior-contingent critique than two of the three low vitality lessons. These inconsistencies are not necessarily surprising, given the small sample size of the study.

Although a relatively clear distinction between high and low vitality lessons emerged when considering the observational results in total, a more nuanced examination of the data suggests that the characteristics of low and high vitality students’ lessons are likely to vary in somewhat idiosyncratic ways according the context of any given lesson as well as particular student and teacher pairings. For example, the lesson for high vitality student one involved a great deal of teacher talk, to which the student responded with performances rather than words. Because of this particular student-teacher dynamic, the number of actions of both student and teacher is very high. In contrast, the lesson for high vitality student three emphasized the mental aspects of performance, and thus included the most elaboration questions and also the least performance trials. Despite the difference in teaching and learning styles, both students reported experiencing high vitality following their lesson.

### Rapport

The composite scores of the rapport ratings indicated much more evidence of rapport between students and teachers of the high vitality lessons (*M* = 14.33, min = 14, max = 15) compared to the low vitality lessons (*M* = 5.67, min = 4, max = 7). Moreover, the means, minimum values, and maximum values of the ratings for each of the three rapport items were higher for the lessons of the high vitality student as compared to those of the low vitality students. The means of the ratings for the mutual attentiveness dimension were *M* = 1.33 (min = 1, max = 2) for the low vitality lessons and *M* = 5.00 (min = 5, max = 5) for the high vitality lessons. The mean ratings for the positivity dimension were *M* = 2.67 (min = 1, max = 4) and *M* = 4.67 (min = 4, max = 5) for the low and high vitality lessons, respectively. The ratings of the coordination dimension were, similarly, disparate with *M* = 1.67 (min = 1, max = 2) for the low vitality lessons and *M* = 4.67 (min = 4, max = 5) for the high vitality lessons.

The proximity ratings for the high vitality students’ lessons were quite high, all videos were scored as a “5-Teacher is near the student all or nearly all of the time.” These teachers either sat or stood with the student for the duration of the lesson, rarely moving away unless to retrieve an item or hear the student play from a different perspective for a brief time. This resulted in an overall impression that the student and teacher were working together toward common goals in the lesson. However, the proximity ratings for the low vitality students were more mixed, with ratings ranging from “1-Teacher is far from student all or nearly all of the time” to “3-Teacher spends roughly equal time near to and far from student.” The teachers of the low vitality students clearly spent less time near their students. The teachers in each of the low vitality lessons tended to use barriers in their studio space to separate them from their students (i.e., desk, chair, piano). Although similar barriers were present in the teachers’ of the high vitality students lesson spaces, they did not stop them from being in proximity to the students.

### Contextual Narrative Summaries

#### Low Vitality Student Lessons

A lack of mutually understood goals between student and teacher seemed to be the defining feature of the low vitality students’ lessons. All of the students reporting low vitality were asked to choose what material they would like to play in the lesson that day. Consequently, the lesson activities appeared to arise primarily in response to what the teacher was hearing in the moment and did not appear to reflect an overarching, *a priori* plan for the students’ development, or an approach that took the students’ perspective in a more meaningful way. Student improvement was unclear in these lessons because they tended to focus upon very small sections of music. The teachers in these lessons typically did not give the students the opportunity to perform passages they worked on within broader contexts of their repertoire, and often did not ask students to repeat the passage after providing feedback. The students tended to be passive in terms of their response to feedback and the teachers tended to be businesslike in their approach. As is evident in the behavioral analyses, the low vitality students performed less and received less feedback than their high vitality peers. Relatedly, the pace of the lessons tended to be slow.

#### High Vitality Student Lessons

All of the high vitality students’ lessons included clearly articulated goals set by both teacher and student. A sense of structure was evident in these lessons since students understood that they were expected to play previously devised exercises and routines. The overall tone of these lessons was friendly and each lesson included some degree of conversation about the student’s life outside of their lessons. The students were quite active throughout the lesson and responded to their teachers’ feedback consistently, with either verbal responses or through additional performance attempts. The teachers were attentive and engaged throughout the lessons and as such, the pacing tended to be rapid. Teachers’ critiques of student playing were typically presented in a neutral tone and the teachers responded enthusiastically when the students overcame challenges. Performance skill improvement was clear in all three lessons.

## Discussion

The purpose of this study was to investigate relationships among student and teacher behavior, rapport, and students’ experiences of subjective vitality in the context of university-level applied performance lessons. The teaching behaviors we observed reflected findings of previous research on expert teachers (e.g., Duke and Simmons, 2006; Parkes and Wexler, 2012; Blackwell, 2018): high expectations were evident, students performed a great deal, students were stopped frequently and asked to repeat materials with higher accuracy, teachers modeled plentifully, teachers engaged in side-coaching while students played, critical feedback was plentiful, and praise tended to occur when students made progress. This was not surprising, given that all of the teachers had clear reputations in their respective fields as expert pedagogues.

We were particularly interested in examining whether teacher behaviors varied according to their students’ reports of subjective vitality immediately following their video-recorded lesson. Vitality is a construct that encompasses the subjective experiences of enthusiasm, aliveness, and energy available to the self (Ryan and Frederick, 1997; Ryan and Deci, 2008). Self-determination theorists have demonstrated that the subjective state of wellbeing described in the construct of vitality is more likely to emerge when individuals are in autonomy-supportive (rather than controlling) social contexts and when they have their basic needs of autonomy, competence, and relatedness met (Nix et al., 1999; Deci et al., 2006).

The high vitality students’ lessons were characterized as beginning with mutually agreed upon goals and objectives, suggesting a sense of structure to the lesson which has been shown to be important for autonomy support (e.g., Reeve et al., 2002; Ryan and Deci, 2017). This may seem counterintuitive at first, because it might be supposed that structure would impose limitations on students’ choices and ability to freely and flexibly allow the lesson to progress in a way that is most suited to the students’ learning. However, it appears here, as with other studies (e.g., Sierens et al., 2009; Jang et al., 2010; Vansteenkiste et al., 2012) that structure communicates common expectations to students and provides a framework within which both parties can freely engage. The established structure of the lesson meant that it was predictable for both the teacher and the student, and they were able to focus on the music without having to negotiate new expectations or agenda for each lesson. The uncertainty around a lack of structure may be one factor associated with the outcomes of the low vitality group.

Relatedly, one of the main features of autonomy-supportive teaching is often described as “providing choices” (e.g., Reeve et al., 1999; Evans, 2015; Ryan and Deci, 2017). But in this case it was the low vitality students who were provided with more choice. It may that the established structure and predictability of the lessons, with a teacher who took the perspective of the student and provided instruction accordingly, was more engaging than an uncertain lesson with no agenda, that began with a difficult and less meaningful choice. Indeed, this is a finding that resonates with research on school classroom motivation and engagement (e.g., Assor et al., 2002; Aelterman et al., 2019). The high vitality students played much more frequently and addressed a relatively larger amount of exercises and repertoire — lesson attributes that likely contributed to the relatively quicker pace observed. These lesson features were also more likely to contribute to a student’s sense of competence, given that they created more opportunities for students to experience accomplishment. Low vitality students identified few goals and objectives and a relatively limited breadth of topics, had smaller amounts of repertoire addressed, and slower pacing—features that could have also negatively impacted the students’ feelings of competence. For example, low vitality students expressed frustration and asked their teachers for help more often.

The teachers of the high vitality students asked more questions that promoted critical thinking and served as informal formative assessments, whereas the teachers of the low vitality students asked questions that revealed they were not entirely aware of their student’s recent activities or performance status. Questions that support student inquiry and independence are much more likely to be autonomy-supportive than questions that serve to orient the teacher to the students’ status (e.g., Reeve and Jang, 2006). The teachers of the high vitality students were also more likely to ask the students about their lives outside of lessons and share their own musical histories — reflecting the connection between basic needs-fulfilling experiences and increased subjective vitality (Ryan and Deci, 2017). These behaviors can support a students’ sense of relatedness since they seem to reflect the teachers’ desire to connect with their students personally as well as their concern for mentoring their student to develop a sense of the professional culture and an identity as a musician. The greater frequency of laughter occurring during the high vitality students’ lessons may suggest that a stronger personal bond was present between those student-teacher pairs as well.

The differences in feedback observed between the lessons of the high and low vitality students were also compelling. The teachers of the high vitality students were generally more forthcoming with information about performance, provided more behaviorally-contingent critique – though with a neutral as opposed to a critical tone, and were more likely to comment on the specific aspects of their students’ playing that improved during the lesson. These same teachers were also more frequently observed providing cognitive models for their students, likely fulfilling these students’ needs for competence (Ryan and Deci, 2017). Overall, the high vitality students received more plentiful, specific, and task-relevant feedback than their low vitality peers and were also given more insight into the ways their teachers conceptualized and solved problems via informational lecture and cognitive modeling. These lesson attributes are indicative of an environment that is supportive of a student’s sense of competence and autonomy (Reeve and Jang, 2006).

There were also important differences between the high and low vitality lessons in terms of observed rapport. The high vitality lessons were rated higher on all dimensions of the researcher-developed rapport scale, suggesting that there may be a relationship between rapport and subjective vitality. Additionally, a number of the observed behaviors in the high vitality lessons were indicative of strong rapport, including teachers sharing their personal musical history, more questioning suggesting an investment in developing student’s independent musicianship, more recognition of specific progress and student effort, and the prevalence of laughter. The narrative contextual commentary revealed the presence of conversations about the student’s lives outside of their lessons, suggesting an investment in the students beyond their musical progress. These findings are consistent with previous research suggesting that strong interpersonal relationships may be key to effective teaching and learning in studio music instruction (Nerland and Hanken, 2002; Kurkul, 2007; Montemayor, 2008; Wexler, 2008; Clemmons, 2009/2010; Hyry-Beihammer, 2010; Creech, 2012; Nolan, 2012; Blackwell, 2018). As noted earlier, they also reflect the connection between needs-fulfilling experiences (particularly in relation to the need for relatedness in this case) and subjective vitality (Ryan and Deci, 2017).

Teacher-student proximity was also more prevalent in the high vitality students’ lessons, suggesting that proximity may also be associated with higher observed rapport, although additional research is necessary to investigate this claim. The teachers of the high vitality students tended to spend the majority of the lesson side-by-side with their student. It should be noted that the typical setup of studio music instruction includes a number of potential physical barriers, such as pianos, desks, and music stands, that may result in greater physical distance between student and teacher. Thus, it would seem that in the low vitality lessons, teachers allowed these physical objects to become barriers, although they were not necessarily hiding or using them purposefully as barriers. In contrast, the teachers in the high vitality lessons appeared to be aware of deliberately not allowing these potential barriers to separate them from their students. While infrequent, there were also instances of touch in the high vitality lessons that are associated with immediacy, such as high-fives or pats on the shoulder, which are typically associated with affirmation or reassurance. Conversely, there were no such instances in the low vitality lessons. As issues of proximity and immediacy have been very rarely studied in the music education literature (Kurkul, 2007; Roseth, 2018), there are many opportunities for additional research in this area.

### Limitations and Suggestions for Future Research

The results of this study should be viewed in light of its limitations. While this study provided an in-depth analysis of six lessons, the sample size is not sufficient to make broadly generalizable claims about the practices of studio music instruction. Future research might incorporate larger samples of both students and teachers. In future work, for larger-scale quantitative analyses, researchers may wish to consider establishing validity and reliability of proximity measures. For future qualitative work, member-checking may also be a worthwhile dimension of analysis. Given that the measure of subjective vitality in our study is self-report, it could be valuable for researchers to collect complementary sources of data (e.g., peer, parent, or teacher reports) for the sake of triangulating the students’ reports. We note here the potential reliability issues associated with single-item measures rated by potentially subjective researchers. Future work might use more independent observers on multi-item scales to address this limitation. And further qualitative work could investigate any potential discrepancies between ‘objective’ raters and the participants’ own experiences, either as a member checking procedure to ensure the portrayal of the participant’s subjective experience, or as an exploration of the discrepancy as a research question in and of itself. Moreover, in-depth qualitative study of how contextual factors, teachers’ pedagogical style, and students’ learning style are related to the unique combinations of behaviors that appear in low and high vitality students’ lessons might reveal a variety of distinct profiles of autonomy supportive teaching.

Only a single lesson was observed for each student, representing a 1-day snapshot in a presumably multi-year student-teacher relationship. Future studies might observe lessons over an extended period of time to better understand how student vitality could be related to lesson behaviors, particularly in relation to instructional scaffolding over time. Researchers could also investigate students’ activities between lessons to better understand successful student-teacher relationships. Because students spend so much of their time and effort outside of weekly lessons, understanding students’ self-directed learning tendencies and what they bring to this relationship could be key in developing a more nuanced understanding of successful teaching and learning. Additionally, because it is possible that investigations with younger, less experienced music learners may yield markedly different results.

Although the data from this study suggest relatively clear associations between students’ reports of vitality and particular attributes of studio music instruction, it is not possible to draw causal inferences from this study. Because vitality can be influenced by a number of factors, it is important to consider factors external to the lesson for both students and teachers. While somewhat independent of physiological state, vitality is also impacted by other stressors, such as diet, exercise, and issues experienced apart from studio music instruction experiences. Questionnaires that consider these external factors, as well as other factors salient to the lesson context (i.e., the amount of time they have been working with their teacher, the time of day and setting of the lesson) might help provide further context in future research. It is also possible that students experiencing various levels of vitality may bring out certain kinds of behaviors in their teachers, and thus future research might incorporate self-reports of teachers as well as students. We did not take into account performance ability or expertise in relation to the analysis, so it could simply be that the high vitality students were such because they were satisfied performing at a much higher level, and they were more likely to have an easier, more pleasant relationship with their teacher.

Finally, the quantitative ratings of expert teaching strategies, proximity, rapport, and the contextual narrative summaries were all ratings made by the researchers involved, who each had knowledge of the students’ vitality ratings. Notwithstanding this limitation, the present study suggests differences, in some cases stark differences, between low and high vitality students that suggest the viability of larger scale research. Such research could involve larger numbers of studio music lessons and blindness to the dependent variables on the part of the researchers.

### Implications

The findings of the present study suggest a number of profitable avenues for pedagogical practice. It would be worthwhile to experiment with how students react to the behaviors that seem to differentiate low vs. high vitality student experiences in practice. Teaching interventions using the behaviors associated with the high vitality students’ lessons would be relatively easy to implement, and may help to foster high vitality in students. Relatively straight-forward changes in questioning, feedback, and modeling behavior are not particularly difficult to implement; these are also behaviors that novice teachers would be able to learn to use with little struggle. For example, novice teachers could view videos of successful pedagogues or discuss teaching cases that could help them understand these attributes by seeing them in action. The use of teaching cases (live via online technologies or video recorded) has been suggested as a potentially viable method for introducing students to real-life teaching scenarios in previous literature (i.e., West, 2012; Blackwell and Roseth, 2018). Alternatively, teachers could engage in reflection exercises to look for these behaviors by observing videos of their own teaching and assessing the degree to which they use what appear to be impactful teaching practices.

Our findings also point to the importance of identifying a good student-teacher match in studio music instruction, as the ability to work efficiently and effectively is particularly important in a one-to-one environment. It is possible that the low vitality students were less optimally matched to their teachers in regard to personality, goals, or other factors. Additional research is needed to understand how best to match students with teachers (and vice versa) in studio music instruction and how effective student-teacher matching may facilitate learning.

## Conclusion

Music learning requires intensive focus and effort for extended periods, often including moments of frustration and demanding patience for delayed gratification. Vitality is emblematic of the energy necessary to focus attention, persist on challenges, and practice deliberately, suggesting that high vitality may serve to support positive learning outcomes over time. Conditions that enable vitality are also those that allow someone to reap the benefits of their environment and help to internalize the values of the profession, which could assist in the development of a musician’s overall identity (Ryan and Deci, 2017). Students high in vitality are also more likely to exhibit intrinsic motivation and the integration of external motivation (Kasser and Ryan, 1996) – attributes all teachers would like to see in their students. Examining what sorts of teaching conditions lead to students’ feelings of subjective wellbeing can lead to valuable improvements in studio music instruction in the profession.

## Data Availability Statement

The datasets for this article are not publicly available because sharing the video data would violate the anonymity of study participants. Requests to access the datasets should be directed to JB, blackw@hawaii.edu.

## Ethics Statement

The studies involving human participants were reviewed and approved by Human Research Ethics Advisory Panel at University of New South Wales, Sydney. The patients/participants provided their written informed consent to participate in this study.

## Author Contributions

JB and PM conceived of the initial idea and study design. PE and GM provided feedback on the study design. PE, PM, and GM oversaw data collection. JB and PM carried out data analysis and interpretation. All authors discussed the results and contributed to the final manuscript.

## Conflict of Interest

The authors declare that the research was conducted in the absence of any commercial or financial relationships that could be construed as a potential conflict of interest.
