# Supplementary material for: Student Vitality, Teacher Engagement, and Rapport in Studio Music Instruction
Source: Front Psychol. 2020 May 21;11:1007. doi: 10.3389/fpsyg.2020.01007 (PMC7253673; doi:10.3389/fpsyg.2020.01007)
Supplement: Supplementary file 1 [file Data_Sheet_1.docx]

**Appendix A**

- Teacher Behaviors
  - Teacher Question – Elaborate
    - Teacher asks student to elaborate verbally on a topic
  - Teacher Question – Check
    - Teacher does a quick check-in that student is aware of what’s happening that moment
  - Teacher Question – Goals
    - Teacher asks student about their goals related to the lesson
  - Teacher Question – Orienting
    - Teacher asks students question about where they are in their studies (implying they are unaware of student’s history to some degree)
  - Discussion – Practicing
    - Teacher explicitly describes methods for students to use to practice outside of the lesson
  - Discussion – Personal, unrelated
    - Teacher discusses a personal experience unrelated to the musical goals of the lesson
  - Discussion – Personal, music
    - Teacher discusses a personal experience related to the musical goals of the lesson
  - Feedback – Low recognition of general progress
    - Low magnitude teacher feedback recognizing progress made during the lesson that is not specifically tied to a particular objective
  - Feedback – High recognition of general progress
    - High magnitude teacher feedback recognizing progress made during the lesson that is not specifically tied to a particular task
  - Feedback – Low recognition of specific progress
    - Low magnitude teacher feedback recognizing progress made during the lesson that is specifically tied to a particular task
  - Feedback – High recognition of specific progress
    - High magnitude teacher feedback recognizing progress made during the lesson that is specifically tied to a particular objective
  - Feedback – Low person-directed praise
    - Low magnitude teacher praise pertaining to some personal attribute of the student (rather than a task)
  - Feedback – High person-directed praised
    - High magnitude teacher praise pertaining to some personal attribute of the student (rather than a task)
  - Feedback – Person-directed critique
    - Teacher feedback critiquing some personal attribute of the student (rather than a task)
  - Feedback – Behavior-contingent critique
    - Teacher feedback critiquing a particular task the student demonstrated
  - Feedback – Behavior-contingent praise
    - Teacher feedback praising a particular task the student demonstrated (without reference to how they performed earlier – i.e., not “progress”)
  - Feedback – Normative critique
    - Teacher feedback critiquing a student by comparing them to others
  - Feedback – Normative praise
    - Teacher feedback praising a student by comparing them to others
  - Feedback – Instructional information
    - Teacher provides neutral generic information with no overt sense of critique or praise
  - Modeling – Instrument
    - Teacher demonstrates for the student by playing their instrument
  - Modeling – Vocal
    - Teacher demonstrates for the student by singing
  - Modeling – Cognitive
    - Teacher explains the way they think/conceptualize a particular musical issue for the student
  - Modify passage to reduce complexity
    - Teacher alters the task the student is attempting to perform by simplifying it in some way
  - Gestural side coaching during performance
    - Teacher physically gestures/moves to direct the student while the student is playing
  - Vocal side coaching during performance
    - Teacher sings/speaks along with the student while the student is playing
  - Teacher touches student – Rapport
    - Teacher touches students in a friendly manner
  - Teacher touches student – Physical manipulation
    - Teacher touches student to adjust their body for some aspect of performance
- Student Behaviors
  - Student Performance
    - Student plays their instrument
  - Student Question – Request for assistance
    - Student asks teacher for help about a performance task
  - Student Question – Request for feedback
    - Student asks teacher for feedback regarding a performance trial
  - Student Talk - Off task
    - Student engages in discussion of topics unrelated to musical goals of the lesson
  - Student Talk – Negative expression
    - Student expresses negativity verbally (e.g., frustration, anger, sadness)
  - Student Talk – Positive expression
    - Student expresses positivity verbally (e.g., pride, joy, happiness)
  - Student Comment – Description
    - Student makes a generic comment about the activities of the lesson, often answering a teacher’s question
- Teacher/Student Behaviors
  - Laughing
    - Either teacher or student laughs out loud

**Appendix B**

| ***Note Sheet for Lesson Context Narratives*** | |
| --- | --- |
|  | |
| *Topic* | *Notes* |
| **Tone during the lesson**  *How does the lesson begin… ? i.e., disciplinary, business-like, welcoming, inviting, friendly…* |  |
| **Priority goals for lesson**  *What general performance or musicianship goals are the focus of the lesson, if any… ?*  *i.e., preparing a recital, upcoming jury, reflecting on recent performance…* |  |
| **Style of student engagement**  *What is the student’s general approach to engagement in the lesson… ? i.e., distracted, hostile, disengaged, passive, enthusiastic, focused, active, stalling…* |  |
| **Style of teacher engagement**  *What is the teacher’s general approach to engagement in the lesson… ? i.e., distracted, hostile, disengaged, passive, enthusiastic, focused, active, stalling…* |  |
| **Sense of progress as a result of instruction during the lesson**  *Is the student improving as a result of the teachers’ instruction…?i.e., responding to feedback and directives* |  |
| **Other** |  |
